# Supplementary material for: The application of sudoscan for screening microvascular complications in patients with type 2 diabetes
Source: PeerJ. 2022 Mar 14;10:e13089. doi: 10.7717/peerj.13089 (PMC8929165; doi:10.7717/peerj.13089)
Supplement: Supplemental Information 3 [file peerj-10-13089-s003.pdf]

Thresholds for score of nephropathy (Nephro score) values

March 2016

Background: Diabetic kidney disease (DKD), indicated by albuminuria and/or reduced glomerular filtration rate (GFR) predicts end-stage renal disease, cardiovascular disease and all-cause mortality. Due to its insidious nature and lack of overt symptoms until the advanced stages, regular laboratory testing is needed to detect DKD and its progression. Guidelines recommend screening for DKD on an annual basis by measuring serum creatinine and urine albumin excretion. Yet, many patients are not screened due to poor disease awareness, testing costs and/or lack of infrastructure and personnel to support screening especially in low resource areas or busy clinic settings.
Sweat glands are innervated by autonomic nerves (mainly sympathetic) that, due to their length, can be damaged very early in several disease processes including diabetes. Since autonomic neuropathy reflects microvascular damage due to hyperglycemia and related metabolic perturbation, the severity of sudomotor dysfunction may serve as a surrogate for other microvascular complications including nephropathy.
Results issued from Sudoscan recordings are presented as Electrochemical Skin Conductances (ESC); when ESC and age are combined, a score for higher risk of nephropathy can be calculated. This nephro score has been validated in several studies by comparison with usual tests for DKD diagnosis.
To facilitate interpretation of the nephro score results in a patient, thresholds established from data issued of multiple clinical studies are proposed on the model of thresholds proposed for ESC.

Origin and justification of threshold values:

Study	Reference test used as reference	Population	Nephro score	Sensitivity	Specificity
Osaki	Estimated glomerular filtration rate (EGFR) < 60 ml/min/1.73m^2	(n=100, patients with type 2 diabetes, 50 with CKD, 50 without)	**55**	94%	78%
Luk	Estimated glomerular filtration rate (EGFR) < 60 ml/min/1.73m^2	(n=2933, patients with type 2 diabetes)	**53**	77%	63%
Freedman	Estimated glomerular filtration rate (EGFR) < 60 ml/min/1.73m^2	(n=276, patients with diabetes)	**44**	69%	73%

In a study by Osaki the mean nephro score in patients without DKD (mean EGFR 116±28 ml/min/1.73m^2) was 62±11 (median EGFR 104.00 (98–114) ml/min/1.73m^2) while it was 44±9 in patients with CKD (median EGFR 37 (29–45) ml/min/1.73m^2).
In a study by Luk the mean nephro score in patients without DKD (mean EGFR 116±28 ml/min/1.73m^2) was 59±15 while it was 46±12 in patients with CKD (mean EGFR 42±14 ml/min/1.73m^2).
In a study by Freedman the mean nephro score in patients without DKD (mean EGFR 88 ml/min/1.73m^2) was 62 while it was 47 in patients with CKD (mean EGFR 30 ml/min/1.73m^2).

Conclusion: In these studies performed in different populations and using different and recognized risk factors there was a significant risk of DKD evidenced for a nephro score < 55

Thresholds for nephro score: > 60 (green); 60-40 (yellow); < 40 (orange/red)

References:

Ozaki R, Cheung KK, Wu E, Kong A, Yang X, Lau E, Brunswick P, Calvet JH, Deslypere JP, Chan JC. A new tool to detect kidney disease in Chinese type 2 diabetes patients—comparison of EZSCAN with standard screening methods. Diabetes technology & therapeutics 2011;13(9):937-43
Luk AO, Fu WC, Li X, Ozaki R, Calvet JH, Chung H, Wong R, So WY, Chow CC, Chan JC. The Clinical Utility of SUDOSCAN in Chronic Kidney Disease in Chinese Patients with Type 2 Diabetes. Plos ONE. 2015 Aug 13;10(8):e0134981.
Freedman BI, Smith SC, Bagwell BM, Xu J, Bowden DW, Divers J. Electrochemical skin conductance in diabetic kidney disease. Am J Nephrol 2015;41(6):438-447.
